# Identifying Biomarkers to Predict the Prognosis of Biliary Atresia by Weighted Gene Co-Expression Network Analysis

**DOI:** 10.3389/fgene.2021.760182

**Published:** 2021-11-25

**Authors:** Meng Kong, Bo Xiang

**Affiliations:** Department of Pediatric Surgery, West China Hospital, Sichuan University, Chengdu, China

**Keywords:** LECT2, prognosis, native liver survival, biliary atresia, WGCNA

## Abstract

The prognosis of children with biliary atresia (BA) after Kasai operation remains difficult to predict, and liver fibrosis is closely related to the prognosis of children with BA. We aimed to find biomarkers for native liver survival (NLS) prediction by weighted gene co-expression network analysis (WGCNA). The biological processes and signal pathways that biomarkers involved in were further analyzed by bioinformatics. Quantitative Real-time PCR, Western blot and immunohistochemistry was performed to detect biomarkers expression. The relationship of biomarkers with clinicopathological characteristics of BA was also investigated. LECT2 was overexpressed or knockdown in LX-2 cells, and the expression of fibrogenic genes such as *a*-SMA and COL1A1 was quantified. We found that LECT2 mRNA expression was higher in BA liver tissues compared with normal liver tissues. Bioinformatics showed that LECT2 mainly played a fibrosis-promoting role in the development in BA by regulating bile acid metabolism and promoting inflammatory response. LECT2 immunohistochemistry scores of BA children were higher than control group (*p* = 0.001). Survival analysis revealed that LECT2 high expression is an unfavorable prognostic factor for native liver survival in BA patients. Additionally, the high LECT2 expression was an independent prognostic factor affecting native liver survival (HR 3.702, 95%CI:2.085–6.575, *p* = 0.001). LECT2 modulates TGF-β mediated *a*-SMA and COL1A1 expression in LX-2 cells. siRNA-LECT2 inhibits the expression of *a*-SMA and COL1A1 in LX-2 cells. Overexpression of LECT2 resulted in an increase in *a*-SMA and COL1A1 expression. Knockdown of LECT2 inhibits the proliferation and increase apoptosis in activated LX-2 cells. LECT2 may act as a new prognostic biomarker for native liver survival in BA patients.

## 1 Introduction

Biliary atresia (BA) is a severe, progressive obstructive biliary disease that occurs in infancy and is a common cause of neonatal jaundice (Hartley et al., 2009). Inflammatory bile duct obstruction and liver fibrosis are the key factors affecting the survival of children with BA. Since the Kasai procedure was performed in 1959, the prognosis of BA children has gradually improved (Chardot et al., 1999). However, despite the Kasai procedure, more than 60% of children still need liver transplantation to save their lives due to recurrent cholangitis and cholestatic cirrhosis after surgery (He et al., 2021). Furthermore, Kasai surgery does not stop the progression of liver fibrosis, and about 70–80% of children with liver fibrosis continue to progress, affecting long-term prognosis (Bijl et al., 2013). How to prolong the survival time of native liver in BA children and delay liver transplantation is an urgent clinical problem.

In recent years, the development of bioinformatics has greatly contributed to the understanding of diseases and helped to analyze the role of individual genes in the disease process at the genomic level as a whole. The weighted gene co-expression network analysis (WGCNA) can screen gene modules closely related to diseases by analyzing the correlation between genomic and clinical information, thus providing a basis for further experimental studies (Luo et al., 2015). In this study, we analyzed the gene expression profile of patients with BA by WGCNA algorithm and screened that the key genes associated with hepatic fibrosis in biliary atresia is LECT2 (Leukocyte Cell Derived Chemotaxin 2). LECT2 is a protein coding gene and has a neutrophil chemotactic activity. LECT2 is involved in many immune processes, such as regulation of sepsis, regulation of hepatocellular carcinoma cells, and regulation of neurological diseases (Zheng et al., 2013).

Continued progressive liver fibrosis is one of the most important factors affecting the prognosis of BA, and we need to prevent or slow down the occurrence of liver fibrosis in our clinical work. In reviewing the literature, no data was found on the association between LECT2 and native liver survival of biliary atresia patients. In this study, we analyzed the relationship of LECT2 with native liver prognosis in BA patients, and identified the potential prognosis value for BA patients.

## 2 Methods

### 2.1 Microarray Data of Biliary Atresia and Bioinformatics Analysis

The gene expression profiles of biliary atresia were obtained from the Gene Expression Omnibus (available at http://www.ncbi.nlm.nih.gov/geo) database. GSE46960 and GSE15235 dataset were included in our study. The GSE15235 included 26 fibrosis biliary atresia liver tissues and 17 inflammatory biliary atresia liver tissues. The GSE46960 included 64 biliary atresia liver tissues, 14 diseased control liver tissues and seven normal liver tissues. GSE15235 was used for WGCNA analysis, and the annotation information of the chip was obtained from the GPL570 platform. The “Affy” package in R language (version 4.1) was used for preprocessing of raw data (Sásik et al., 2002), and the “WGCNA” package in R language was used for the construction of weighted gene co-expression networks and module identification, and the modules that were significantly correlated with clinical phenotypes were extracted (Langfelder and Horvath 2008). Firstly, the gene expression matrix correlation coefficients were weighted to make the interaction relationships between genes conform to the scale-free distribution. Then the genes were classified and genes with similar expression patterns were divided into a module. Then the characteristics of the modules were studied to identify the modules most associated with BA liver fibrosis. Finally, the network regulatory relationships between genes within the modules were explored, and hub genes were extracted using Cytoscape and visualized for protein-protein interactions (PPI) analysis (Killcoyne et al., 2009). Gene Set Enrichment Analysis (GSEA) was used to analyze the potential role of LECT2 in the development of BA. The mRNA expression data of BA patients were obtained from the above GEO data, and GSEA was used to annotate the role of LECT2 in BA. GSEA v4.1.0 for Windows (http://www.gsea-msigdb.org/gsea/index.jsp) was downloaded, and gene sets were obtained from Molecular Signatures Database v7.4 for analysis and visualization of results with *p* < 0.05 and false discovery rate (FDR) < 0.05 were statistically significant (Subramanian et al., 2005).

### 2.2 Patient Selection and Tissue Sampling

Total 205 children with biliary atresia after Kasai operation admitted to our hospital between January 2015 and December 2020 were selected as the case group. Inclusion criteria: The age of surgery was less than 180 days and the liver tissue of the child was pathologically diagnosed as biliary atresia. Exclusion criteria: Those who no clear pathological diagnosis. Data was collected including gender, age at surgery, laboratory examination and Pediatric End-stage Liver Disease (PELD) score (Chang et al., 2018). During surgery, a 5 mm-sized piece of liver tissue was excised from the lower edge of the right lobe of the liver. The liver tissue was placed in formalin solution and will be used for immunohistochemical staining. Forty children with hepatoblastoma were selected, and normal liver tissue adjacent to the tumor were taken as the control group. The collection of samples for this study was approved by the Biomedical Research Ethics Committee (No.1082) and informed consent was obtained from the parents of the patients. All the BA children were followed up after surgery, with a median follow-up time of 18 months (3–60 months). A native liver survival (NLS) status within 5 years post-Kasai operation was also recorded in the study.

### 2.3 Immunohistochemical Analysis

Liver tissues were sectioned, dewaxed, rinsed, incubated with 0.3% H_2_O_2_ for 5 min at room temperature. Slides were incubated with 5% BSA in PBS for 30 min at 37 °C to block non-specific binding sites, then incubated with appropriate primary antibodies (anti-human LECT2 antibody, 1:150, ab119429) overnight at 4 °C, followed by horseradish peroxidase anti-mouse IgG antibody for 1 h. The slides were then incubated with DAB substrate kit for color development. Each pathological section was observed in five randomly selected fields under a high-powered microscope. Positive expression of LECT2 was defined as the presence of brown or yellowish-brown granular material in the cytoplasm. The score of LECT2 expression was determined by the proportion of positive cells and the degree of staining. According to the proportion of positive cells: 0 points ≤5%; 1 point 6-25%; 2 points 25–50%; 3 points 50–75%; four points ≥75%. According to the degree of staining: 0 points: no staining; one point: light staining, slightly higher than the background color; two points: moderate staining; three points: strong staining. The total score was obtained by adding the proportion of positive cells and the staining degree score (-) 0–1 point (+) two to three point (++) four to five point (++++) six to seven point, where 0–3 was low expression and four to seven was high expression.

### 2.4 Cell Culture

The LX-2 cell line was inoculated with DMEM medium supplemented with 10% fetal bovine serum, 100 U/ml penicillin, and 100 mg/ml streptomycin, and cultured in an incubator at 37°C, 70%–80% humidity, and 5% CO_2_. When the cell growth density reached 80–90%, the cells were digested with 2 ml trypsin and passaged. TGF-*β* is the cytokine with the strongest effect in stimulating hepatic stellate cells (HSC) activation and secretion of extracellular matrix (ECM), so TGF-*β* was used in this study to stimulate LX-2 cells in order to establish a liver fibrosis model.

### 2.6 RNA Isolation and Quantitative Real-Time Polymerase Chain Reaction (qRT-PCR)

Total 20 biliary atresia liver tissues and 10 normal liver tissues were used to RNA Isolation. Total RNA was extracted from liver tissue using Trizol, and total RNA was used for cDNA synthesis through NovoScript® first Strand cDNA Synthesis SuperMix (Novoprotein Scientific Inc. China). PCR amplification was performed using SYBR® Premix Ex Taq™ kit and data analysis was performed by the 2^−ΔΔCt^ method. LECT2 forward primer: GCT​GGT​CTG​ATT​TCT​ACC​GCA; LECT2 reverse primer: TCC​AGC​AGA​GCA​CAA​GAT​GTC.

### 2.7 Plasmid Construction and RNA Interference

The design and synthesis of LECT2-specific siRNA and pCS2-LECT2 plasmid was done with the assistance of Tsingke Biotechnology Co., Ltd. LX-2 cells were cultured for 24 h and transfected according to the instructions of LipofectamineTM2000 (Invitrogen). The medium was changed after 6h transfection and TGF-*β* was added at a concentration of 10 ng/ml and continued to be cultured for 48h. The sequences of oligonucleotides used are as follows:

LECT2-siRNA (human):5′- UUU​UGA​GUG​GGU​AUC​AAC​CAG-3’; Negative scrambled siRNA:5′- GUU​GAU​ACC​CAC​UCA​AAA​AGG-3’;

LECT2-clone-F: 5′- TTG​CAG​GAT​CTG​CCA​CCA​TGT​TTT​CCA​C-3’;

LECT2-clone-R: 5′- ATT​GAT​GCC​TCG​AGC​CCG​GGT​TAC​AGG​TAT​G-3’.

#### 2.7 Western Blot

Whole cell proteins were lysed with RIPA buffer containing 1% PMSF, centrifuged at 15,000 r/min for 30 min, and the supernatant was collected. The protein was extracted using the protein extraction kit according to the instructions, and the protein concentration was determined by the BCA kit. 20 μg of protein was separated by 10% SDS-PAGE, and transferred to PVDF membrane. The membrane was closed with 5% skim milk for 1 h at room temperature, and diluted LECT2 primary antibody, *a*-SMA primary antibody, Col1*α*1 primary antibody, and *ß*-actin primary antibody were added and incubated overnight at 4°C. TBST washed 3 times, and diluted HRP-labeled secondary antibody was added and incubated for 2 h at room temperature. ImageJ software was used to analyze and calculate the grayscale values.

### 2.8 Cell Proliferation

The cell density of LX-2 cells was adjusted to 4×10^5^ cells/well, and when the cell density reached 80%, 10 ng/ml TGF-β was added and the culture was continued for 24 h. Subsequently, transfection was performed. After 24, 48, 72 and 96 h of incubation, 20 μL of CCK-8 reagent was added to each well. Set the enzyme marker at 450 nm and detect the OD value of the cell CCK-8 mixture for viability analysis.

### 2.9 Cell Apoptosis

According to the manufacturer instruction of Annexin-V-FITC Apoptosis Detection Kit, the LX-2 cell concentration was adjusted to 5 × 10^5^cells/ml, and the cells were resuspended with 400 μL of Binding Buffer. Add 5 μL AnnexinV-FITC staining solution to the above cell suspension, mix well, and incubate for 15min. Add 10 μL PI staining solution and incubate for 8min. Transfer the cell suspension into a flow cytometry plastic tube and perform cell apoptosis analyses on the BD FACSVerse machine.

### 2.10 Statistical Analysis

All data were analyzed by R software (R 4.1.0). The Chi-Square test was used to compare the rates, and the non-parametric test was used to compare the quantitative variables. Survival curves were calculated using the Kaplan-Meier method, and differences were tested with log-rank tests. The Cox proportional risk model was used to determine independent factors based on the variables selected by univariate analysis. The *p* < 0.05 was considered statistically significant.

## 3 Result

### 3.1 Bioinformatics Analysis and Hub Genes

**FIGURE 1:**
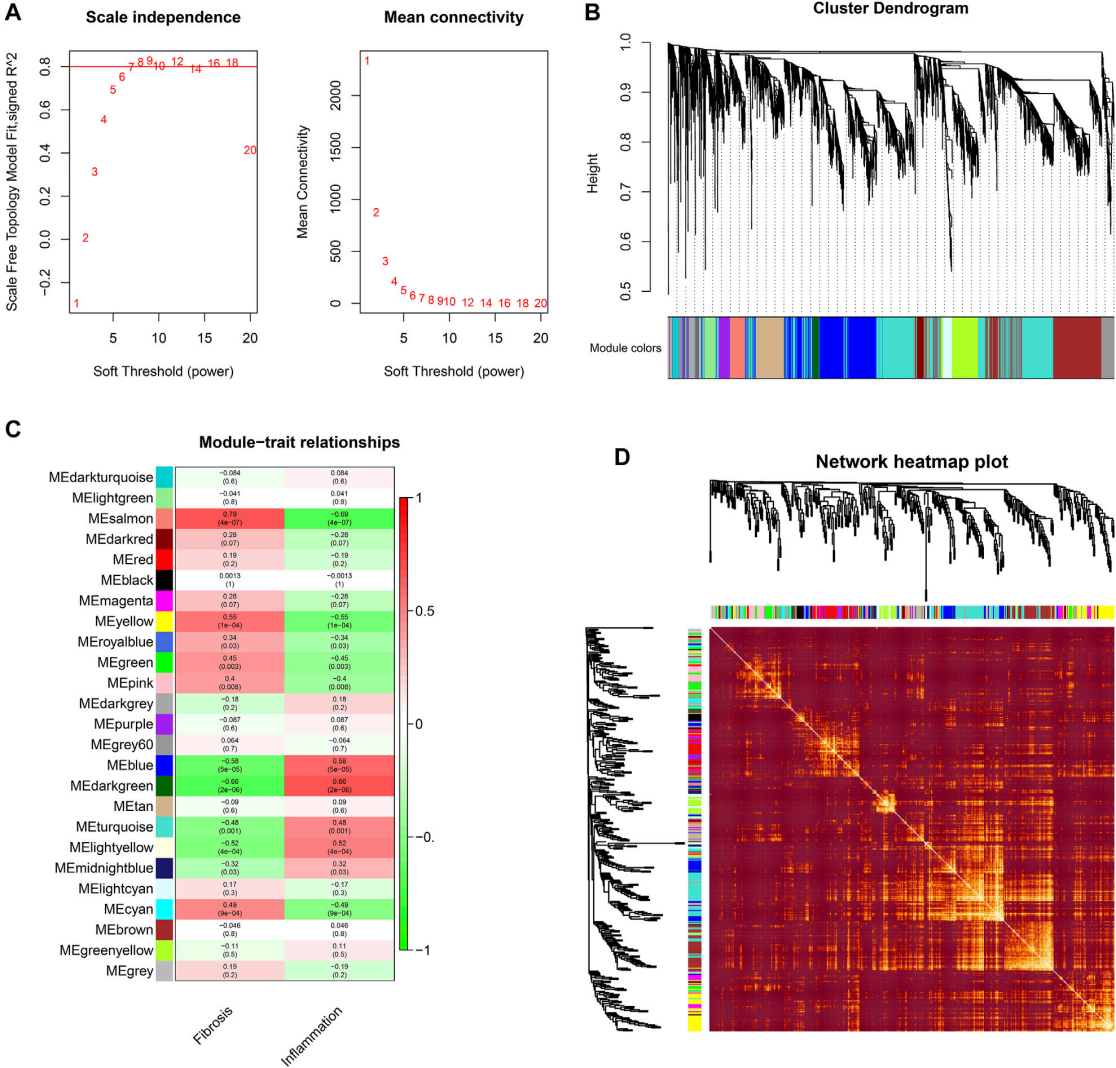
Construction of co-expression modules **(A)** Determination of soft-threshold power **(B)** The cluster dendrogram of genes **(C)** Heatmap of the module-trait relationships **(D)** Network heatmap plots of genes selected for WGCNA construction.

**FIGURE 2:**
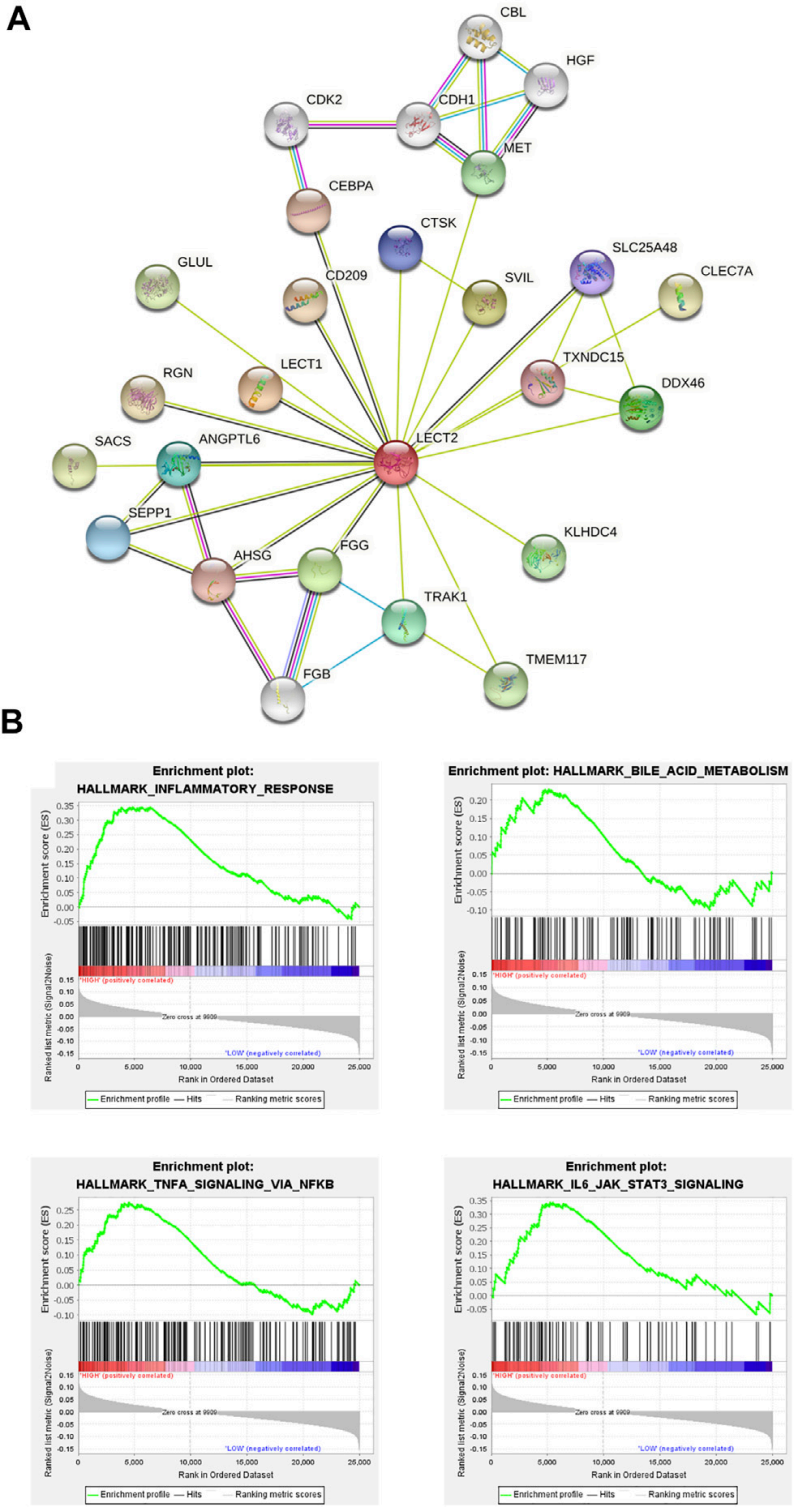
Identification of hub genes **(A)** The protein–protein interaction (PPI) network **(B)** Gene set enrichment analysis.

In this study, when the soft threshold is 7, the *R* ^2^ of Scale Free Topology Model reached 0.8, and the *R* ^2^ tends to be stable (Figure 1A). A total of 24 gene modules and one grey module were obtained, and different modules were assigned different colors, among which the grey module corresponded to those genes that were not included in any module, and the modules with more genes clustered were blue, turquoise and darkred modules (Figure 1B). The correlation between the salmon module and the clinical phenotype was high (r = 0.79, *p* < 0.001), suggesting that the genes in the salmon module were significantly correlated with the clinical phenotype of liver fibrosis (Figure 1C). The hierarchical clustering heat map shows that the correlation between the genes within the modules is high, and it also shows that the modules are not independent of each other, but are also correlated (Figure 1D). The genes in the salmon module were analyzed using Cytoscape, and hub genes were extracted through the plugin MCODE. LECT2 is located at the core of the network (Figure 2A). The results of GSEA analysis showed that LECT2 is involved in BA development mainly through regulation of bile acid metabolism, activation of genes involved in the inflammatory response, activation of TNF-*α* signaling pathway and IL16 signaling pathway (Figure B).

### 3.2 LECT2 Expression in Liver Tissue

**FIGURE 3:**
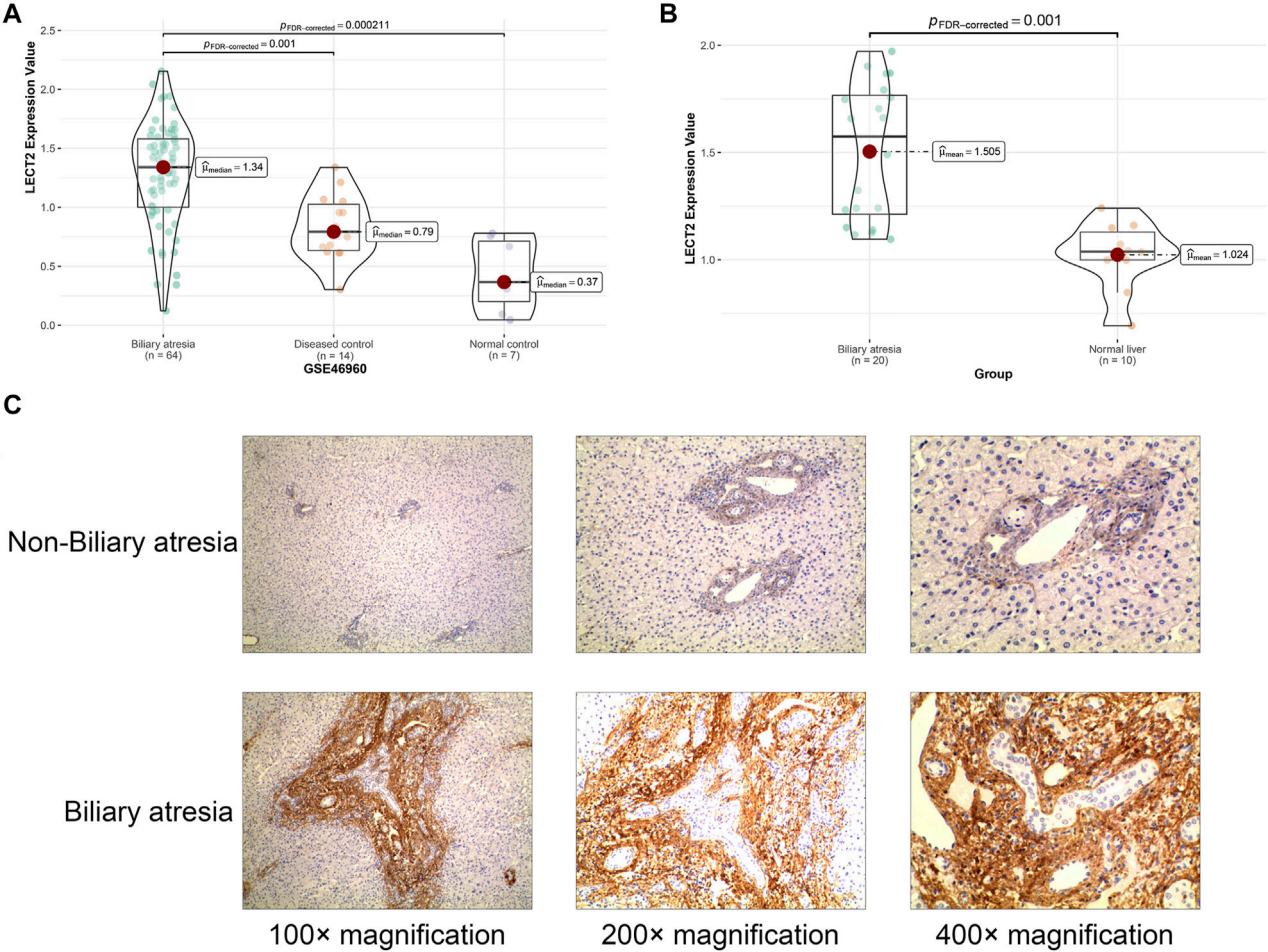
The expression of LECT2 in biliary atresia **(A)** The relative expression of LECT2 in GSE46960 **(B)** The relative expression of LECT2 in clinical specimens **(C)** Immunohistochemistry for LECT2 in biliary atresia and non-biliary atresia groups.

GSE46960 was used to validated the mRNA expression level of LECT2 in biliary atresia. The mRNA expression of LECT2 was found increased in biliary atresia liver tissues (Figure Figure3A). Then, we examined the expression of LECT2 in biliary atresia liver tissues and normal liver by qRT-PCR. The result showed that the expression of LECT2 in BA liver tissues was higher than that in normal liver tissues (Figure 3B). Additionally, immunohistochemistry was used to detect the LECT2 protein expression in BA. Total 205 BA children underwent Kasai surgery in our hospital, including 89 males and 116 females, and all children were followed up from 1 month to 60 months. There were 104 BA patients survived 5 years with native liver after Kasai surgery, with a 5 years native liver survival rate of 54.6% and a median survival time of 32.8 months. Total 101 children underwent liver transplantation or died due to liver function failure. The clinical information of BA was summarized in Table 1. LECT2 was mainly expressed in the cytoplasm of hepatocytes. LECT2 was highly expressed in the cytoplasm of biliary atresia hepatocytes (Figure 3C). LECT2 protein expression in biliary atresia (87.8%) was significantly higher than in control group (7.5%, *p* < 0.01; Table2).

**TABLE 1:** Clinical information of biliary atresia patients.

	Overall	Native liver survival	Native liver death	*p* Value
	(N = 205)	(N = 104)	(N = 101)	
Gender				
Female	116 (56.6%)	60 (57.7%)	56 (55.4%)	
Male	89 (43.4%)	44 (42.3%)	45 (44.6%)	0.854
Age(day)				
Mean (SD)	75.7 (22.2)	74.5 (22.4)	77.0 (22.1)	0.408
Weight(kg)				
Mean (SD)	5.17 (1.08)	5.21 (1.04)	5.12 (1.13)	0.549
Growth failure				
Absence	181 (88.3%)	97 (93.3%)	84 (83.2%)	
Present	24 (11.7%)	7 (6.7%)	17 (16.8%)	0.042
TB(μmol/L)				
Mean (SD)	160 (67.0)	147 (60.6)	174 (70.7)	0.004
DB(μmol/L)				
Mean (SD)	136 (60.5)	128 (60.3)	144 (59.9)	0.058
IB(μmol/L)				
Mean (SD)	24.1 (16.1)	21.5 (14.4)	26.7 (17.3)	0.019
ALT(IU/L)				
Mean (SD)	191 (168)	195 (172)	188 (164)	0.77
AST(IU/L)				
Mean (SD)	328 (411)	344 (514)	311 (268)	0.566
ALP(IU/L)				
Mean (SD)	465 (239)	452 (236)	477 (243)	0.455
GGT(IU/L)				
Mean (SD)	606 (483)	646 (514)	565 (447)	0.23
ALB(g/L)				
Mean (SD)	39.1 (5.39)	39.9 (4.94)	38.2 (5.70)	0.019
LDH(IU/L)				
Mean (SD)	348 (113)	341 (121)	354 (104)	0.416
INR				
Mean (SD)	1.22 (0.486)	1.10 (0.259)	1.35 (0.618)	0.001
FIB(g/L)				
Mean (SD)	2.18 (0.737)	2.23 (0.759)	2.12 (0.715)	0.317
WBC(10^9/L)				
Mean (SD)	12.6 (4.77)	12.5 (4.32)	12.8 (5.21)	0.674
Neutrophil(10^9/L)				
Mean (SD)	4.62 (2.93)	4.28 (2.16)	4.96 (3.53)	0.096
Lymphocyte(10^9/L)				
Mean (SD)	6.54 (3.55)	6.28 (3.33)	6.80 (3.75)	0.291
Monocyte(10^9/L)				
Mean (SD)	0.778 (0.376)	0.774 (0.366)	0.782 (0.388)	0.88
PLT(10^9/L)				
Mean (SD)	413 (171)	417 (174)	409 (168)	0.758
Creatinine(umol/L)				
Mean (SD)	20.9 (8.86)	21.1 (10.7)	20.7 (6.41)	0.743
PELD				
Mean (SD)	8.98 (6.06)	6.51 (4.66)	11.5 (6.30)	0.001
LECT2				
High	109 (53.2%)	29 (27.9%)	80 (79.2%)	
Low	96 (46.8%)	75 (72.1%)	21 (20.8%)	0.001

TB:Total Bilirubin; DB:Direct Bilirubin; IB:Indirect Bilirubin; ALT:Alanine Transaminase; AST:Aspartate Aminotransferase; ALP:Alkaline phosphatase; GGT:γ-Glutamyltransferase; ALB:Albumin; LDH:Lactate dehydrogenase; INR:International normalized ratio; FIB:Fibrinogen; WBC:White blood cell count; PLT:Platelet; PELD:Pediatric End-stage Liver Disease.

**TABLE 2:** Comparison of LECT2 protein expression between biliary atresia liver tissue and control tissue.

Groups	Total	LECT2 high expression		LECT2 low expression		*p* Value
		n	%	n	%	
Biliary atresia	205	180	87.8	25	12.2	
Normal liver	40	3	7.5	37	92.5	<0.01

### 3.3 Correlation of LECT2 Expression in Biliary Atresia and Native Liver Survival

**FIGURE 4:**
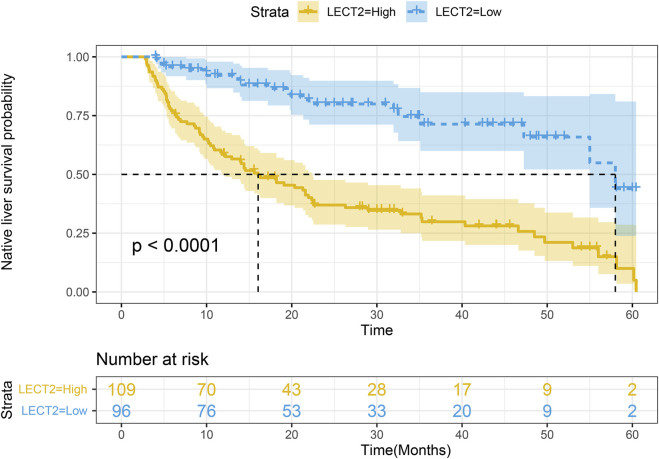
Kaplan–Meier survival curves of native liver survival in biliary atresia.

The children were divided into high LECT2 expression group and low LECT2 expression group. The native liver survival rate of BA children in the low LECT2 expression group was 78.1%. The native liver survival rate of BA children in the high LECT2 expression group was 26.6%. In the low LECT2 expression group, the median survival time after Kasai surgery was 58 months. The median survival time after Kasai surgery was 16 months in the high LECT2 expression group. The Log-Rank test showed a statistically significant difference in the native liver survival rate between the two groups (*p* < 0.001), and the survival rate comparison curves are shown in Figure 4. Based on the results of the univariate analysis, a multivariate COX regression analysis was performed for the seven statistically significant influencing factors: Growth failure, PELD score, TB, DB, IB, INR and LECT2 expression. The high LECT2 expression was an independent prognostic factor affecting native liver survival (Table3). BA patients with high LECT2 expression had a 3.7-times higher native liver mortality risk than patients with low LECT2 expression.

**TABLE 3:** Cox regression analysis of native liver survival.

	Univariable analysis							Multivariable analysis						
Parameters	Hazard ratio (95% CI)						*p* Value	Hazard ratio (95% CI)						*p* Value
Weight(kg)	1.089	(	0.823	—	1.44	)	0.552							
Growth failure	4.129	(	1.215	—	14.036	)	0.023	1.374	(	0.799	—	2.363	)	0.25
TB(μmol/L)	1.003	(	1.001	—	1.006	)	0.008	0.999	(	0.992	*—*	1.007	)	0.845
DB(μmol/L)	1	(	1	—	1.006	)	0.044	0.998	(	0.992	*—*	1.004	)	0.469
IB(μmol/L)	1.012	(	1.001	—	1.023	)	0.036	1.001	(	0.983	—	1.018	)	0.954
ALT(IU/L)	1	(	0.998	—	1.001	)	0.601							
AST(IU/L)	0.999	(	0.998	—	1	)	0.119							
ALP(IU/L)	1	(	0.999	—	1.001	)	0.839							
GGT(IU/L)	1	(	1	—	1.001	)	0.347							
ALB(g/L)	1.006	(	0.962	—	1.052	)	0.798							
LDH(IU/L)	1.001	(	0.999	—	1.003	)	0.237							
INR	1.482	(	1.141	—	1.925	)	0.003	0.357	(	0.085	*—*	1.504	)	0.16
FIB(g/L)	1.078	(	0.771	—	1.508	)	0.661							
WBC(10^9/L)	0.974	(	0.922	—	1.03	)	0.357							
Neutrophil(10^9/L)	1.056	(	0.973	—	1.147	)	0.192							
Lymphocyte(10^9/L)	1.036	(	0.965	—	1.112	)	0.327							
Monocyte(10^9/L)	1.538	(	0.808	—	2.93	)	0.19							
PLT(10^9/L)	1.001	(	0.999	—	1.002	)	0.299							
Creatinine(μmol/L)	1.005	(	0.978	—	1.034	)	0.714							
PELD	1.188	(	1.006	—	1.418	)	0.045	1.019	(	0.985	—	1.054	)	0.276
LECT2	3.462	(	1.713	—	6.993	)	0.001	3.702	(	2.085	—	6.575	)	0.001

TB:Total Bilirubin; DB:Direct Bilirubin; IB:Indirect Bilirubin; ALT:Alanine Transaminase; AST:Aspartate Aminotransferase; ALP:Alkaline phosphatase; GGT:*γ*-Glutamyltransferase; ALB:Albumin; LDH:Lactate dehydrogenase; INR:International normalized ratio; FIB:Fibrinogen; WBC:White blood cell count; PLT:Platelet; PELD:Pediatric End-stage Liver Disease.

### 3.4 LECT2 is Involved in Regulating the Formation of Hepatic Fibrosis in Biliary Atresia

**FIGURE 5:**
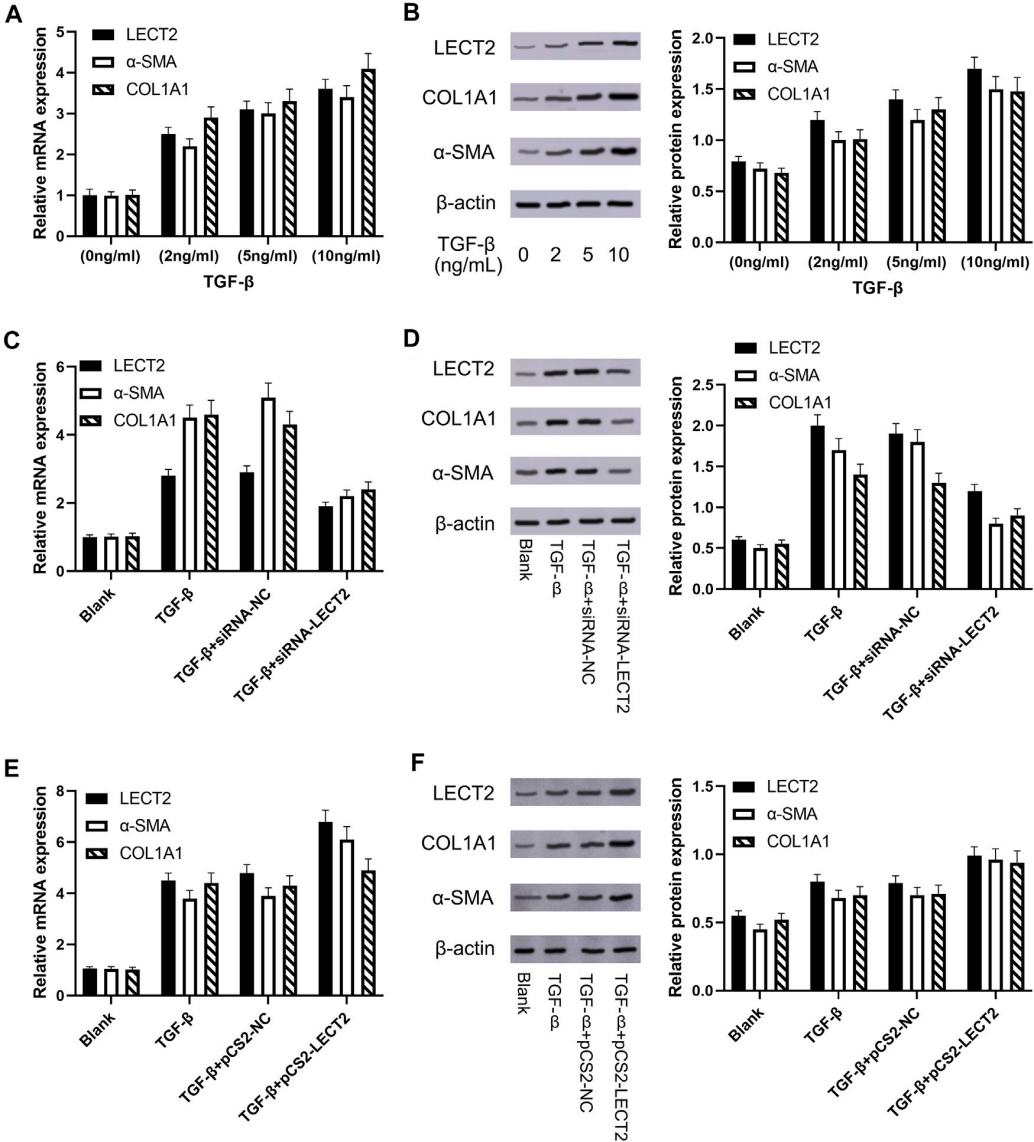
The expression of LECT2, *a*-SMA and COL1A1 in LX-2 cells **(A)** The effect of TGF-*β* on LECT2, *a*-SMA and COL1A1 mRNA **(B)** The effect of TGF-β on LECT2, *a*-SMA and COL1A1 protein **(C)** siRNA-LECT2 inhibits the mRNA expression of *a*-SMA and COL1A1 in LX-2 cells **(D)** siRNA-LECT2 inhibits the protein expression of *a*-SMA and COL1A1 in LX-2 cells **(E)** Overexpression of LECT2 resulted in an increase in *a*-SMA and COL1A1 mRNA levels **(F)** Overexpression of LECT2 resulted in an increase in *a*-SMA and COL1A1 protein levels.

Progressive liver fibrosis is one of the most important factors affecting the prognosis of biliary atresia children, and hepatic stellate cells are the key cells involved in the development of biliary atresia liver fibrosis. TGF-*β* is considered to be an important pro-fibrotic cytokine. We induced LX-2 cell activation with different concentrations of TGF-*β* (2, 5, and 10 ng/ml). Real-time qPCR results showed that the mRNA expression of LECT2, *a*-SMA, and COL1A1 was significantly increased in the experimental group (Figure 5A). Western blot showed that the protein expression of LECT2, *a*-SMA, and COL1A1 was significantly increased in the experimental group (Figure 5B). We used siRNA transient transfection technique to silence LECT2 in LX-2 cells and observed its effect on fibrosis indexes. Real-time qPCR results showed that the mRNA expression of LECT2, *a*-SMA, and COL1A1 were significantly higher in the experimental group after 24 h of TGF-*β* (10 ng/ml) stimulation compared with the normal group. In contrast, the expression of LECT2, *a*-SMA, and COL1A1 was significantly decreased after transfection with siRNA-LECT2 (Figure 5C). The Western Blot results also showed that transfection with siRNA-LECT2 significantly decreased the protein expression levels of LECT2, *a*-SMA, and COL1A1 under the same conditions (Figure 5D). These results suggest that silencing of the LECT2 gene decreased the index of fibrosis in LX-2 cells. To further confirm the pro-fibrotic effect of LECT2, the plasmid pCS2-LECT2 was transfected in LX-2 cells to overexpress LECT2. The result showed that LECT2, *a*-SMA, and COL1A1 protein and mRNA expression were significantly increased after transfection with pCS2-LECT2 compared to the control group (Figures 5E–F). This also indicated that LECT2 could promote the elevation of fibrosis indicators.

### 3.5 The Effect of LECT2 on LX2 Cells Proliferation and Apoptosis

**FIGURE 6:**
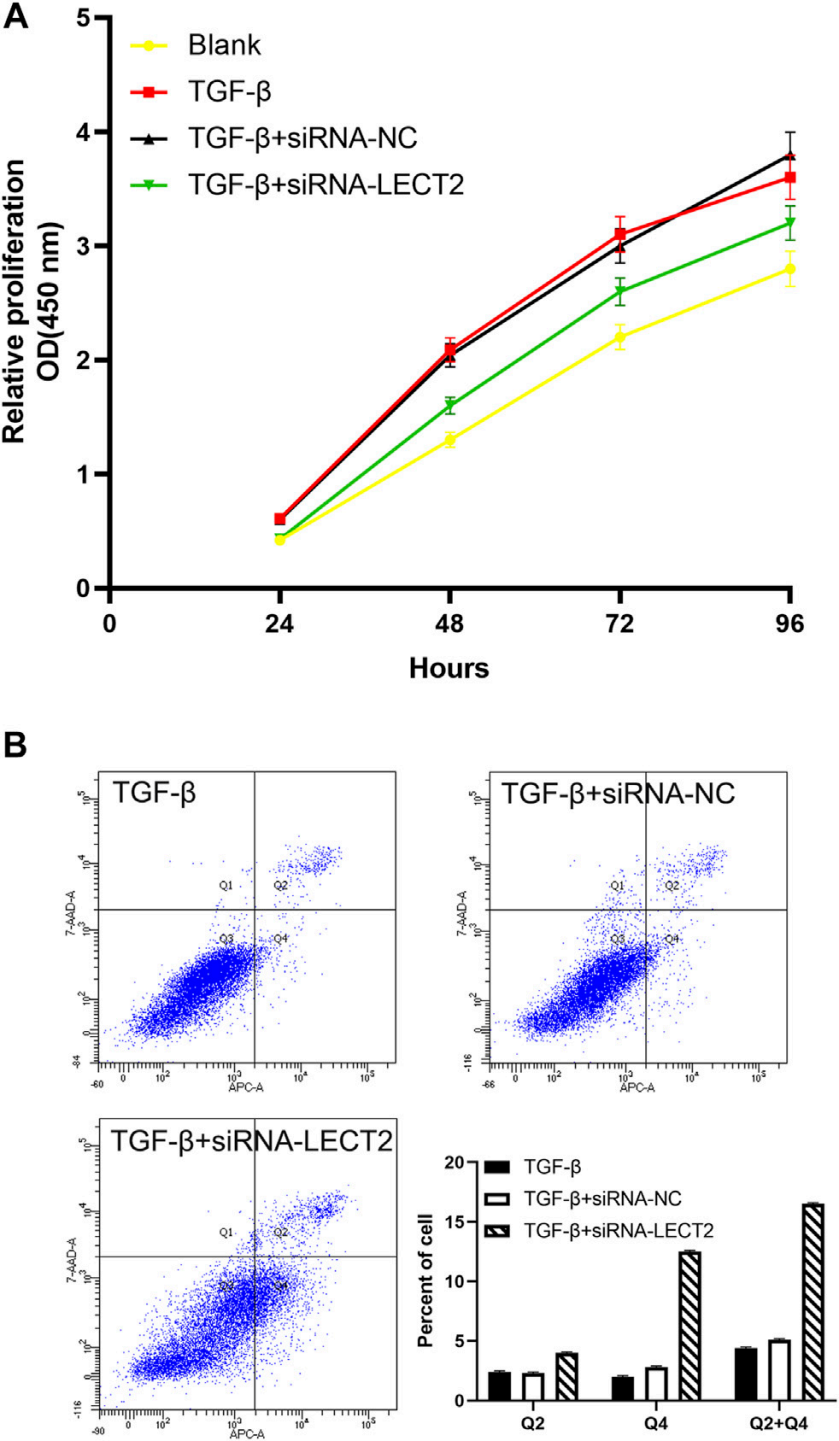
The effect of LECT2 on LX2 cells proliferation and apoptosis **(A)** Knockdown of LECT2 inhibits the proliferation in activated LX-2 cells **(B)** Knockdown of LECT2 increase apoptosis in activated LX-2 cells.

CCK8 results showed that the proliferation of LX-2 cells in the transfected siRNA-IRF3 group was significantly inhibited compared with the control group (Figure 6A). Flow cytometry results showed that the proportion of apoptotic cells was significantly increased in LX-2 transfected with siRNA-LECT2 compared with the control group (Figure 6B).

## 4 Discussion

Biliary atresia is a common cause of pathological jaundice in infants, with complex etiology, and the major pathological changes of BA were collagen deposition and liver tissue fibrosis, which can eventually lead to death of the child (Cielecka-Kuszyk et al., 2021). Studies have shown that liver fibrosis plays an important role in the course of BA and that the process of liver fibrosis does not stop after Kasai surgery in children with BA (Zhou et al., 2016). In this study, using the WGCNA algorithm, we found that the salmon module was most associated with biliary atresia liver fibrosis. The hub gene screened by PPI network was LECT2, which may be a key gene in the development of biliary atresia liver fibrosis.

In our study, the expression of LECT2 in BA liver tissues was higher than that in normal liver tissues. LECT2, a chemotactic factor produced by tissue cells in response to external stimulation, plays an important role in cellular signaling pathways and is a key site in the complex regulation network of liver fibrosis (Xu et al., 2019), but no study has reported the prognosis value of LECT2 in biliary atresia. *In vitro* experiments, LECT2 has specific chemotactic effects on neutrophils, monocytes and macrophages (Okumura et al., 2009). In the pathogenesis of hepatitis, LECT2 is preferentially expressed in hepatocytes in its most basic molecular structure and enters the blood to participate in the inflammatory response. The LECT2-deficient mice treated with concanavalin A resulted in increased liver injury, probably because immune cells such as CD4^+^ T lymphocytes and macrophages were affected by LECT2 deficiency (Saito et al., 2004). Plasma LECT2 levels were positively correlated with visceral fat area and its differential expression was associated with metabolic and dyslipidemia (Tanisawa et al., 2017). When serum glutathione aminotransferase levels reached a peak, the serum LECT2 levels were lowest in patients with acute liver failure, and serum LECT2 levels increased when liver function returned to normal (Sato et al., 2004b). The results of GSEA analysis showed that LECT2 is involved in BA development mainly through regulation of bile acid metabolism, activation of genes involved in the inflammatory response, activation of TNF-α signaling pathway and IL16 signaling pathway. Continued study of these molecular events will contribute to a better understanding of the pathogenesis of BA and potentially translate these findings into the clinical arena. In the future, we will expand the collection of clinical samples and conduct further mechanistic studies at the cellular level and animal level using molecular biological experiments to validate the conclusions drawn from this study.

Our immunohistochemistry result also showed that the proportion of LECT2 high expression in BA children was significantly higher than that of control group, suggesting that LECT2 was highly expressed in BA children and may be involved in the pathogenesis of BA. Previous study showed that LECT2 expression is significantly elevated in nonalcoholic fatty liver disease, and LECT2 induces the development and progression of Nonalcoholic fatty liver disease through the STAT-1 signal pathway (Wang et al., 2021). In lipopolysaccharide or d-galactosamine induced acute liver injury animal model, LECT2 was found to be strongly associated with the prognosis of inflammatory liver disease (Okumura et al., 2017). Regarding the 5 years survival rate without liver transplantation, the 5 years native liver survival rate in Japan is 59.7% (Nio et al., 2003). The 5 years native liver survival rate in England and Wales is 46% (Davenport et al., 2011). The 5 years native liver survival rate after Kasai operation in France is 40% (Chardot et al., 2013). Our finding is consistent with these previous articles. The 5 years native liver survival rate in our hospital is 54.6%, and how to improve the long-term survival of native liver is still the direction we are working on. Several reports have shown that LECT2 may be a biomarker of survival prognosis for patients in various liver disease states. Prior studies that have noted the importance of LECT2 in adult living related donor liver transplantation, serum LECT2 levels decreased immediately after surgery in donors and recipients, suggesting LECT2 is involved in liver regeneration after hepatectomy (Sato et al., 2004a). Previous studies evaluating serum LECT2 observed consistent results on whether LECT2 can be used to predict prognosis of acute liver failure, lower serum LECT2 is associated with better prognosis in adult acute liver failure patients (Slowik et al., 2019). This result is important because LECT2 is a target gene of the Wnt/*β*-catenin pathway and plays a key role in stimulating liver regeneration (Ovejero et al., 2004). LECT2 is also a chemokine for neutrophils and macrophages, which are activated and recruited to the liver during the hepatic recovery phase, and lower serum LECT2 levels may indicate less tissue necrosis and a more rapid remission of the inflammatory response (Antoniades et al., 2012). However, the intrahepatic role of LECT2 may be different than the systemic role of LECT2, and the role of LECT2 in liver tissue may be different from that in serum, which requires further investigation. Our study showed that the native liver survival rate of low LECT2 expression group and high LECT2 expression group were 78.1 and 26.6% respectively, suggesting LECT2 protein expression is associated with the prognosis of BA children after Kasai surgery. A multivariate COX regression analysis indicate that the high LECT2 expression was an independent prognostic factor affecting native liver survival, suggest that LECT2 can be used as an auxiliary indicator to determine the prognosis of BA.

Progressive liver fibrosis is one of the most important factors affecting the prognosis of biliary atresia children, and hepatic stellate cells are the key cells involved in the development of biliary atresia liver fibrosis (Song et al., 2021). TGF-β is considered to be an important pro-fibrotic cytokine. We induced LX-2 cell activation with different concentrations of TGF-*β* (2, 5, and 10 ng/ml). Our results showed that the expression of LECT2, *a*-SMA, and COL1A1 was significantly increased in LX-2 cells. The results suggested that the pro-fibrotic effect of LECT2 may be related to its promotion of hepatic stellate cells activation. CCK8 results showed that transfection with siRNA-LECT2 significantly inhibited LX-2 proliferation, and flow cytometry results showed that siRNA-LECT2 transfection significantly promoted LX-2 cell apoptosis. These results suggest that LECT2 may regulate liver fibrosis by regulating HSC cell proliferation and apoptosis.

There were some limitations in our study. First, this was a single-center retrospective study, but all patients were operated by the same surgical team with standard surgical steps could increase the consistency. Second, the molecular mechanism of LECT2 in the prognosis of BA is unclear, further studies are needed to elucidate the multiple functions of LECT2 in BA.

In conclusion, LECT2 is highly expressed in the BA liver tissues, and the upregulation of LECT2 expression indicates a poor prognosis. The high LECT2 expression was an independent prognostic factor affecting native liver survival. The LECT2 protein might be used as an auxiliary indicator to determine the prognosis of BA children. Detection of LECT2 in liver tissues in BA children may help to select the appropriate time for liver transplantation and evaluate the clinical prognosis.

## Data Availability

The datasets presented in this study can be found in online repositories. The names of the repository/repositories and accession number(s) can be found below: https://www.ncbi.nlm.nih.gov/, GSE15235: https://www.ncbi.nlm.nih.gov/, GSE46960. Other data and materials used during the current study are available from the corresponding author on reasonable request.
